# The transcriptional cortical response to daily torpor in *Phodopus sungorus*

**DOI:** 10.26508/lsa.202603677

**Published:** 2026-07-29

**Authors:** Lukas C Bal, Marcel S Woo, Nicola Rothammer, Elena Haugg, Christina Mayer, Annika Herwig, Manuel A Friese

**Affiliations:** 1 Institute of Neuroimmunology and Multiple Sclerosis, University Medical Center Hamburg-Eppendorf , Hamburg, Germany; 2 Institute of Molecular Endocrinology and Physiology, Ulm University , Ulm, Germany

## Abstract

Torpor is an essential evolutionary strategy that allows mammals to conserve resources under extreme environmental conditions. Although the hypothalamus is a key regulator of systemic acclimatisation to energy scarcity and reduced temperature, it remains unclear how the cortex, with its high metabolic demand, endures this state. Here, we performed RNA sequencing on neuronal and non-neuronal nuclei isolated from the cortex of Djungarian hamsters (*Phodopus sungorus*) across different seasonal and metabolic states. We found that non-neuronal cells primarily adjust during the seasonal transition from a summer to winter phenotype, with differential expression of genes linked to circadian rhythm. In contrast, cortical neurons exhibited major transcriptional changes during daily torpor, marked by increased expression of RNA catabolic pathways. Finally, comparison with published hypothalamic datasets revealed distinct transcriptional programmes between the cortex and hypothalamus. Together, these findings highlight cell type– and region-specific transcriptional changes that preserve CNS integrity during metabolic depression.

## Abstract (precis)

Using nuclei RNA-seq from Djungarian hamster cortex across seasonal and metabolic states, we show non-neuronal cells adapt seasonally through circadian genes, whereas neurons reprogramme during torpor, enriching RNA catabolism and revealing transcriptional divergence from hypothalamus during metabolic depression.

## Introduction

Torpor is an evolutionarily conserved strategy used by endothermic mammals to survive extreme environmental conditions. Based on thermoregulatory properties, animals can generally be divided into ectotherms and endotherms, which differ in their ability to maintain body temperature within a narrow physiological range. Among endotherms, heterothermic species exhibit pronounced temporal reductions in metabolic rate and body temperature, water loss, and other physiological functions, a state referred to as torpor (Rezende & Bacigalupe, 2015). Depending on its duration and seasonal occurrence, torpor can be classified either as daily torpor or as hibernation. Seasonal hibernators typically undergo prolonged bouts of torpor, typically associated with low body temperature, that are periodically interrupted by brief normothermic interbout arousals. In contrast, animals displaying daily torpor reduce metabolic rate and body temperature for shorter periods, usually lasting only several hours, and to a lesser extent (Melvin & Andrews, 2009; Geiser, 2013; Andrews, 2019). In general, in circannual hibernators torpor follows a two-switch model: the first switch involves the transition from the summer to the winter phenotype, thereby preparing the organism for the second switch, namely, the induction of torpor. This process is accompanied by systemic metabolic acclimatisations, including characteristic changes in metabolite composition (Serkova et al, 2007).

One species that exhibits daily torpor is the Djungarian hamster (*Phodopus sungorus*). During this hypometabolic state, body temperature transiently decreases by ∼10–20°C for several hours before returning to normothermic levels during arousal. The development of the winter phenotype is associated with a broad range of physiological and morphological acclimatisations and is primarily regulated by photoperiod-dependent changes in nocturnal melatonin secretion from the pineal gland (Steinlechner & Heldmaier, 1982). The adaptive process involves a substantial decline of food intake and body weight, regression of reproductive organs, and moulting to a white winter fur, and takes ∼12 wk to be largely completed (Scherbarth & Steinlechner, 2010; Cubuk et al, 2016). Once body weight and reproduction have been readjusted to winter state, the animals start to spontaneously show bouts of daily torpor as an additional energy-saving strategy. Daily torpor occurs without any obvious acute energetic challenge, that is, with food and water ad libitum and even under thermoneutral conditions (Figala et al, 1973; Heldmaier & Steinlechner, 1981). Animals spontaneously enter torpor for several hours during the light phase (resting phase), markedly reducing metabolism (80%) and body temperature (>15°C), and then return to normothermia and activity during the night (Figala et al, 1973; Heldmaier et al, 2004; Haugg et al, 2021). Torpor frequency varies between individuals; median incidence equates to 2–3 torpor bouts per week (Haugg et al, 2021). The suprachiasmatic nucleus (SCN), the central circadian pacemaker, relays photic information about day to regulate the timing of torpor onset (Körtner & Geiser, 2000; Ishida, 2009). This principle can be exploited in the Djungarian hamster (*Phodopus sungorus*) to induce a winter phenotype with regular torpor episodes by shifting the day–night cycle from 16:8 h light:dark to 8:16 h light:dark for several months (Scherbarth & Steinlechner, 2010; Haugg et al, 2021). Gene expression analyses in the hypothalamus during torpor have revealed differential expression of genes involved in hormone synthesis, including type 2 iodothyronine deiodinase and fibroblast growth factor 21 (Cubuk et al, 2017). More generally, both transcription and protein synthesis are markedly reduced during torpor (van Breukelen & Martin, 2002; Diaz et al, 2004). In the thirteen-lined ground squirrel (*Ictidomys tridecemlineatus*), this suppression is achieved independently of ambient temperature through hyperphosphorylation of eukaryotic initiation factor 2 alpha (Frerichs et al, 1998). However, it remains unclear how neurons in distinct brain regions adapt during torpor.

Previous studies have compared transcriptomic changes across different brain regions and seasonal states, revealing distinct region-specific regulatory patterns, for example, between the cortex and the hypothalamus (Schwartz et al, 2013). However, the bulk transcriptomic approaches used in these studies cannot resolve gene regulation at the level of specific cell types within complex brain tissue. Addressing this heterogeneity requires cell type–specific profiling, which can be achieved by isolating nuclei and separating neuronal from non-neuronal populations using NeuN-based sorting, followed by sequencing (Kulkarni et al, 2019; Tzec-Interián et al, 2025). This approach enables a more focused analysis of neuronal transcriptional responses.

In this study, we set out to investigate the neuronal stress responses to metabolic and temperature challenges by examining cortical neurons of Djungarian hamsters during torpor. Unlike hypothalamic nuclei, the cortex is not directly involved in initiating systemic metabolic changes, yet it is nonetheless exposed to metabolic depression (Jastroch et al, 2016). Insights from neuropathological conditions such as stroke show that acute metabolic shortage can rapidly lead to neuronal death (Saver, 2006; Kuriakose & Xiao, 2020). Understanding how neurons withstand a programmed metabolic depression during torpor may therefore uncover conserved protective mechanisms against metabolic depression. To address this, we combined nuclei isolation, NeuN-based sorting, and sequencing to generate separate neuronal and non-neuronal transcriptomes from cortical tissue across different torpor states. This approach allowed us to identify neuron-specific transcriptional differences because of daily torpor, contrast them with non-neuronal seasonal responses, and compare our findings with hypothalamic transcriptomic datasets. Our results reveal a neuronal induction of RNA catabolic pathways during torpor and highlight a profound divergence between cortical and hypothalamic transcriptional regulation.

## Results

### Isolation of nuclei from the cortices of the Djungarian hamsters

**Figure 1.**
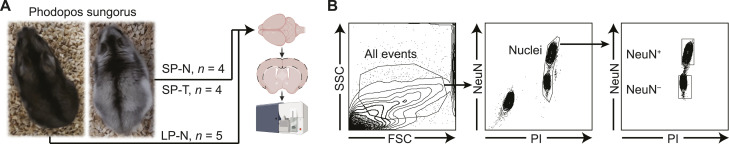
Experimental workflow for isolating neuronal and non-neuronal nuclei. **(A)** Schematic of the experimental design showing *Phodopus sungorus* in summer and winter phenotypes, preparation of the cortical tissue, and subsequent isolation of nuclei. A dashed line indicates the anatomical landmark at which the dissection was performed to isolate the entire isocortical tissue. Neuronal and non-neuronal nuclei were separated by flow cytometric sorting. **(B)** Representative flow cytometry plots of cortical nuclei stained with PI and the neuron-specific nuclear protein NeuN. PI, propidium iodide.

To compare neuronal states and responses to torpor, we collected cortical samples from hamsters in the summer phenotype (long photoperiod normothermia, LP-N) and in the winter phenotype, either on a day without torpor or during torpor (short photoperiod normothermia, SP-N; short photoperiod torpor, SP-T) (Fig 1A). We isolated nuclei from the cortical samples and subsequently separated neuronal from non-neuronal nuclei using fluorescence-activated cell sorting with propidium iodide staining and a fluorophore-labelled NeuN antibody (Fig 1B).

### Enrichment of neuronal transcripts in NeuN-positive nuclei

**Figure 2.**
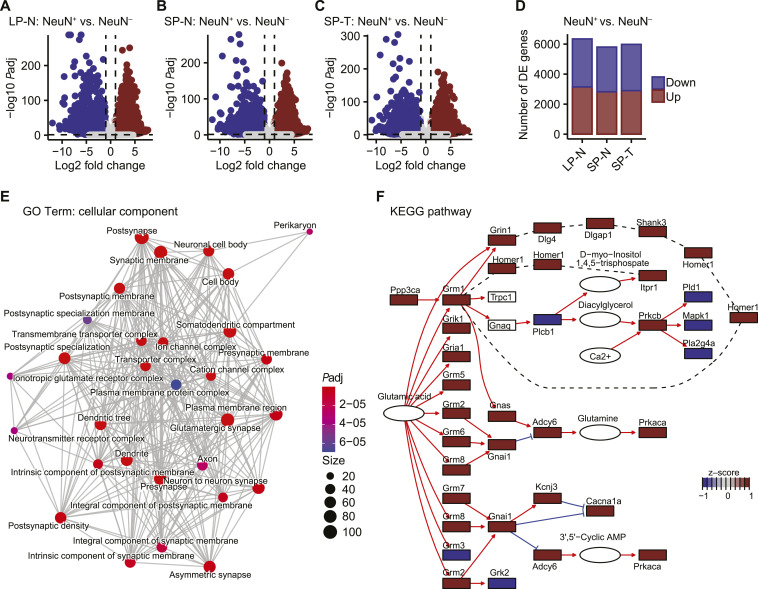
Enrichment of neuronal transcripts in NeuN^+^ nuclei. **(A, B, C)** Volcano plots of DEGs comparing NeuN^+^ and NeuN^−^ nuclei in (A) LP-N, (B) SP-N, and (C) SP-T. **(D)** Number of DEGs detected across metabolic states in NeuN^+^ and NeuN^−^ populations, separated by up- and down-regulation. **(E)** GO enrichment analysis of genes with increased expression in NeuN^+^ nuclei, showing overrepresented cellular component terms. **(F)** KEGG pathway analysis highlighting glutamatergic signalling, with contributing genes colour-coded by the Z-score. LP-N, *n* = 5; SP-N and SP-T, *n* = 4. LP-N, long photoperiod normothermia; SP-N, short photoperiod normothermia; SP-T, short photoperiod torpor.

We next performed bulk RNA sequencing on NeuN-positive (NeuN^+^) and NeuN-negative (NeuN^−^) nuclei to compare transcriptional responses between neuronal and non-neuronal populations. We also aimed to characterise neuronal transcriptional profiles between seasonal and metabolic states. Differential expression analysis between NeuN^+^ and NeuN^−^ nuclei in LP-N, SP-N, and SP-T revealed numerous differentially expressed genes (DEGs), consistent with the well-established transcriptional distinction between neuronal and non-neuronal populations (Fig. 2A–C; Zeisel et al, 2018; Tran et al, 2021). We observed a well-balanced distribution of genes showing increased and decreased expression when comparing NeuN^+^ and NeuN^−^ nuclei across metabolic states (LP-N, SP-N, and SP-T) (Fig 2D).

**Figure S1.**
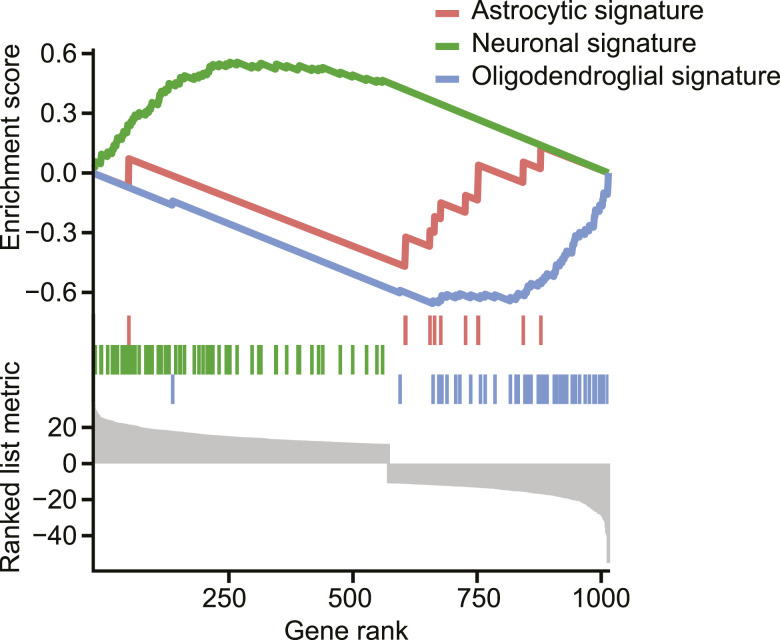
Neuronal gene signatures are enriched in isolated neuronal nuclei. GSEA testing astrocytic, neuronal, and oligodendroglial signatures in genes differentially expressed between isolated NeuN^+^ and NeuN^−^ nuclei. Gene signatures were obtained from Cahoy et al (2008).

To validate the separation of neuronal and non-neuronal nuclei, we performed gene ontology (GO) enrichment analysis on DEGs identified in the NeuN^+^ population. This analysis revealed significant enrichment of neuron-specific pathways and cellular components, including “neuronal cell body,” “glutamatergic synapse,” and “postsynaptic density” (Fig 2E), confirming the successful isolation of neuronal nuclei. To further probe the functional identity of the NeuN^+^ transcriptome, we conducted KEGG pathway analysis (Kanehisa et al, 2017). Given that glutamate is the principal excitatory neurotransmitter in the mammalian brain, we focused on genes associated with glutamatergic signalling— a hallmark of neuronal function. This analysis revealed significant enrichment of multiple genes involved in this pathway, further supporting the neuronal identity of the NeuN^+^ population (Fig 2F). Furthermore, we used a publicly available dataset (Cahoy et al, 2008) to generate neuronal, astrocytic, and oligodendroglial signatures and performed gene set enrichment analysis (GSEA) using genes differentially expressed between the NeuN^+^ and NeuN^−^ fractions. Neuronal signatures showed strong positive enrichment, whereas astrocytic and oligodendroglial signatures were negatively enriched in the NeuN^+^ fraction, corresponding to their enrichment in the NeuN^−^ fraction (Fig S1).

In summary, these findings demonstrate a robust enrichment of neuron-specific transcripts in NeuN^+^ nuclei and confirm the effectiveness of our approach in separating neuronal from non-neuronal cell populations in Djungarian hamster cortices.

### Neuronal transcriptional response only occurs in SP-T

**Figure 3.**
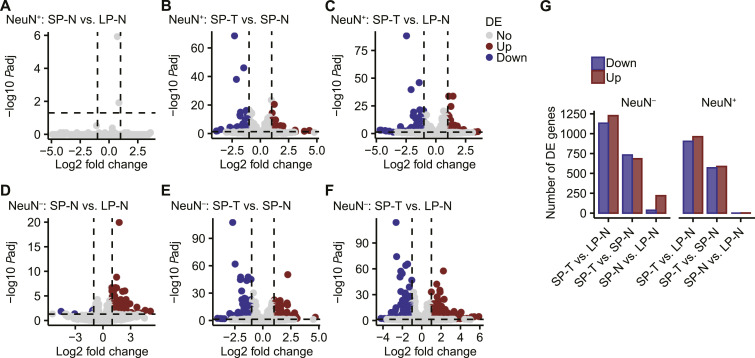
Neuronal transcriptional response only occurs in SP-T. **(A, B, C)** Volcano plots of DEGs in NeuN^+^ nuclei comparing (A) SP-N versus LP-N, (B) SP-T versus SP-N, and (C) SP-T versus LP-N. **(D, E, F)** Volcano plots of DEGs in NeuN^−^ nuclei for the same comparisons: (D) SP-N versus LP-N, (E) SP-T versus SP-N, and (F) SP-T versus LP-N. **(G)** Summary of DEG counts across conditions in NeuN^+^ and NeuN^−^ populations, with colours indicating the direction of regulation. LP-N, *n* = 5; SP-N and SP-T, *n* = 4. LP-N, long photoperiod normothermia; SP-N, short photoperiod normothermia; SP-T, short photoperiod torpor.

After confirming the successful enrichment of neuronal transcripts, we next investigated transcriptional differences across metabolic states. We compared neuronal transcriptomes between SP-N, LP-N, and SP-T, identifying significant sets of DEGs (Fig 3A–C). Notably, we detected no significant transcriptional changes between SP-N and LP-N in neurons, whereas both groups showed numerous DEGs when compared to SP-T.

We then analysed the non-neuronal population across the same seasonal and metabolic states (Fig 3D–F). Again, the fewest DEGs were detected between SP-N and LP-N. However, unlike neurons, some transcriptional differences were present between these two conditions in the non-neuronal population. To summarise, we quantified the number of genes showing increased and decreased expression in both neuronal and non-neuronal populations across seasonal and metabolic states (Fig 3G).

In conclusion, our results suggest that transcriptional differences in cortical neurons are specifically induced under SP-T conditions, supporting the hypothesis that acute metabolic challenges exert a stronger effect on neuronal gene expression in the cortex than the seasonal transition from a summer to winter phenotype.

### Non-neuronal cells show regulation in circadian rhythm and metabolism

**Figure 4.**
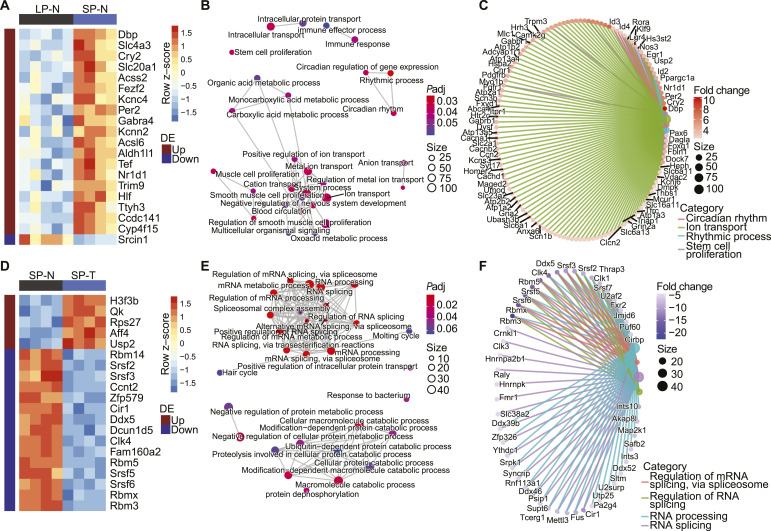
Non-neuronal cells show regulation in circadian rhythm and metabolism. **(A)** Top 20 DEGs in NeuN^−^ nuclei comparing LP-N and SP-N, with bars indicating up- or down-regulation. **(B)** GO enrichment analysis showing overrepresented biological processes among DEGs from (A). **(C)** Circular gene–pathway network plot illustrating functional clustering of DEGs of (A). **(D)** Top 20 DEGs in NeuN^−^ nuclei comparing SP-T and SP-N, with bars indicating up- or down-regulation. **(E)** GO enrichment analysis of DEGs from (D). **(F)** Circular gene–pathway network plot illustrating functional clustering of DEGs from (D). LP-N, *n* = 5; SP-N and SP-T, *n* = 4. LP-N, long photoperiod normothermia; SP-N, short photoperiod normothermia; SP-T, short photoperiod torpor.

To further examine transcriptional changes in non-neuronal populations, we compared LP-N and SP-N samples and visualised the top 20 significantly DEGs (Fig 4A). Among these were key circadian regulators, including *Dbp*, *Per2*, and *Cry2* (Price et al, 1998; Griffin et al, 1999; Ueda et al, 2005; Yoshitane et al, 2019; Patke et al, 2020). GO-term enrichment analysis revealed significant overrepresentation of biological processes related to circadian rhythm and metabolism (Fig 4B). The circular gene–pathway network plot further illustrated functional clustering of DEGs within categories such as circadian rhythm, rhythmic processes, ion transport, and stem cell proliferation (Fig 4C). Although all samples were collected at the same zeitgeber time (ZT) to minimise circadian influences, we observed robust photoperiod-dependent transcriptional changes, consistent with previous reports (Johnston et al, 2005), suggesting that the seasonal transition from a summer to winter phenotype in non-neuronal cells involves reprogramming of circadian and metabolic pathways.

We next examined transcriptional changes associated with the shift from normothermia to hypothermia within the short photoperiod (SP) group (Fig 4D). Notably, we observed differential expression of genes involved in alternative splicing, including *Srsf2*, *Srsf3*, *Srsf5*, and *Srsf6* (Busch & Hertel, 2012; Zheng et al, 2020); and key regulators of RNA stability and metabolism, such as *Ddx5* and *Qk* (Beuck et al, 2012; Xing et al, 2019; Chen et al, 2021). GO-term enrichment analysis confirmed overrepresentation of pathways related to RNA processing, RNA splicing, and macromolecule catabolism (Fig 4E). The corresponding circular gene–pathway network plot showed clustering of top DEGs within categories such as regulation of mRNA splicing via the spliceosome, RNA processing, and RNA splicing (Fig 4F).

In summary, our data indicate that in non-neuronal cells, the summer-to-winter transition primarily affects circadian and metabolic regulation, whereas the normothermia-to-hypothermia transition predominantly impacts RNA metabolism and processing.

### Genes associated with RNA catabolism showed increased expression during torpor

**Figure 5.**
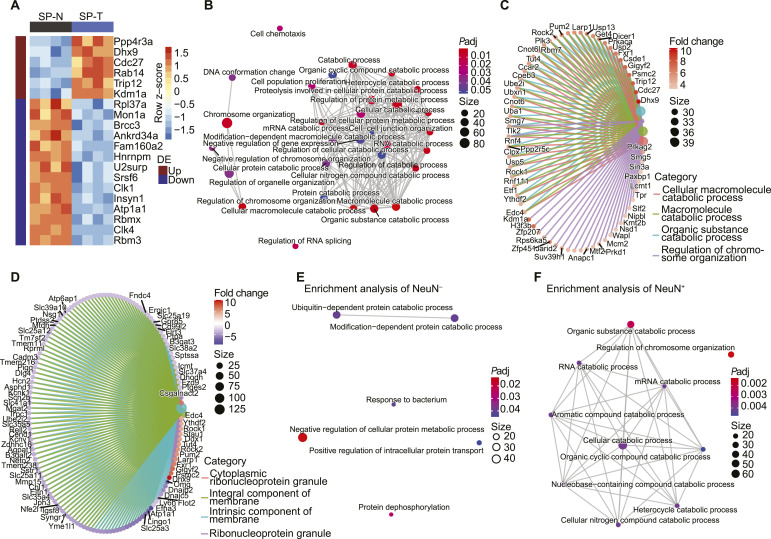
Genes associated with RNA catabolism showed increased expression during torpor. **(A)** Top 20 DEGs in NeuN^+^ nuclei comparing SP-T and SP-N, with bars indicating up- or down-regulation. **(B)** GO enrichment analysis of DEGs from (A) in the category “biological process.” **(C)** Circular gene–pathway network plot of DEGs from (A) within the category “biological process.” **(D)** Circular gene–pathway network plot of DEGs from (A) within the category “cellular component.” **(E, F)** GO enrichment analysis of DEGs in the category “biological process” in (E) NeuN^−^ nuclei and (F) NeuN^+^ nuclei. All groups, *n* = 4. SP-T, short photoperiod torpor; SP-N, short photoperiod normothermia.

We next compared DEGs in neurons between SP-N and SP-T, focusing on the top 20 genes (Fig 5A). Among these, *Dhx9*, a multifunctional RNA helicase involved in transcriptional regulation, splicing, and genome stability, showed increased expression during torpor (Fuller-Pace, 2006; Aktaş et al, 2017). In contrast, *Rbmx*, a key RNA-binding protein implicated in neuronal-specific splicing and chromatin-associated RNA regulation, exhibited decreased gene expression (Nasim et al, 2003; Heinrich et al, 2009; Elliott et al, 2019). GO-term enrichment analysis of the category “biological process” revealed significant regulation of categories such as “RNA catabolic process,” “regulation of RNA splicing,” and “chromosome organisation” (Fig 5B). To further illustrate the functional context of these DEGs, we generated a circular gene–pathway network plot for the term “biological process,” highlighting gene contributions to specific functional categories (Fig 5C). A corresponding analysis was conducted for the term “cellular component,” showing spatial associations of the same gene set (Fig 5D). Comparing enrichment analyses between NeuN^−^ and NeuN^+^ populations revealed only a few enriched terms in NeuN^−^ cells, but numerous significant categories in NeuN^+^ neurons (Fig 5E and F). Notably, NeuN^+^ cells consistently showed enrichment of pathways related to RNA catabolism.

Together, these findings indicate that normothermia-to-hypothermia transition induces transcriptional changes in neurons characterised by increased expression of genes involved in RNA catabolism.

### The cortical response to torpor strongly differs from the hypothalamus

**Figure 6.**
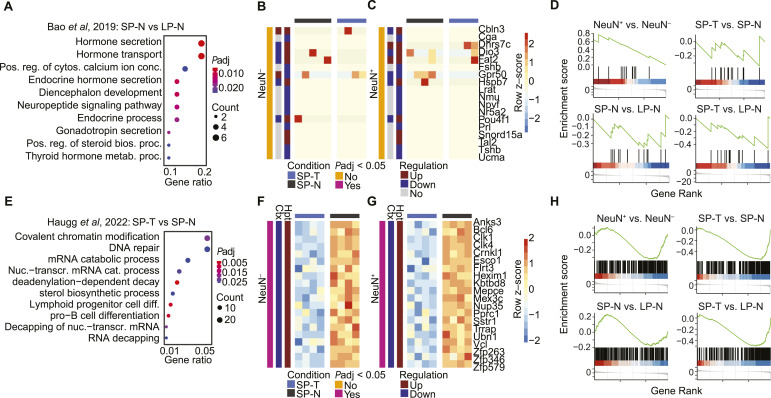
Cortical response to torpor strongly differs from the hypothalamus. **(A)** GO enrichment analysis of hypothalamic DEGs reported by Bao et al (2019). **(B, C)** Expression of the top hypothalamic genes reported by Bao et al (2019), examined in the cortical dataset for (B) NeuN^+^ and (C) NeuN^−^ nuclei. Bars indicate the direction and significance of regulation. **(D)** GSEA testing cortical DEGs against hypothalamic gene sets from Bao et al (2019). **(E)** GO enrichment analysis of hypothalamic DEGs comparing SP-T and SP-N, reported by Haugg et al (2022). **(F, G)** Expression of the top differentially expressed hypothalamic genes reported by Haugg et al (2022), examined in the cortical dataset for (F) NeuN^+^ and (G) NeuN^−^ nuclei. Bars indicate the direction and significance of regulation. **(H)** GSEA testing cortical DEGs against hypothalamic gene sets from Haugg et al (2022).

Finally, we compared our cortical transcriptional responses with those previously reported in the hypothalamus. We reanalysed a published RNA-sequencing dataset examining hypothalamic gene expression between long photoperiod (LP) and SP conditions (Bao et al, 2019). GO enrichment analysis of DEGs from that study revealed strong regulation of terms associated with hormone secretion and endocrine processes. This is consistent with the role of the hypothalamus as a central regulator of hormonal output and systemic acclimatisation such as metabolic down-regulation during torpor (Fig 6A).

When we examined the expression of the top DEGs from the Bao et al study in our cortical data (SP-T versus SP-N), none were significantly altered in either NeuN^+^ or NeuN^−^ populations (Fig 6B and C). GSEA likewise showed no significant overlap with hypothalamic gene sets (Fig 6D).

We also incorporated a second dataset comparing hypothalamic transcriptomes between SP-T and SP-N (Haugg et al, 2022). Reanalysis of these DEGs revealed significant enrichment of pathways related to chromatin modification, mRNA catabolism, and other RNA-associated processes (Fig 6E). Comparing these hypothalamic DEGs with our NeuN^+^ and NeuN^−^ populations revealed that many of the same genes were differentially expressed, but changes were consistently inverted; however, the direction of change was consistently inverted, as genes up-regulated in the hypothalamus during SP-T were down-regulated in the cortex (Fig 6F and G). This opposite regulation was also evident in GSEA, which showed strong de-enrichment when comparing, for example, “SP-T versus SP-N″ contrasts between our dataset and the hypothalamic data (Fig 6H).

Together, comparison with publicly available datasets demonstrates that transcriptional regulation during torpor in the cortex differs markedly from that in the hypothalamus.

## Discussion

In this study, we identified distinct transcriptional regulation in cortical neuronal and non-neuronal cells depending on seasonal and metabolic states. Cortical neurons altered their transcriptional profile only under torpid conditions (SP-T), but not during the seasonal transition (SP-N versus LP-N). This suggests that cortical neurons are less involved in the seasonal transition itself, which is primarily controlled by the hypothalamus, and instead respond predominantly to acute metabolic and temperature-related challenges. In contrast, the non-neuronal population, inducing astrocytes, oligodendrocytes, and microglia, exhibited pronounced seasonal transcriptional reprogramming.

As described above, the transition from the summer to the winter phenotype represents the first step of the two-switch model and enables the subsequent induction of transient torpor (Serkova et al, 2007; Melvin & Andrews, 2009). Torpor itself likely constitutes a substantial metabolic challenge for cortical neurons, as neuronal integrity critically depends on stable energy homeostasis. The pronounced transcriptional response observed in non-neuronal cells during the transition from LP-N to SP-N may therefore indicate that these cells contribute to establishing a metabolic environment that supports neuronal resilience during the transient hypometabolic state of torpor. This interpretation is consistent with the well-established supportive functions of glial cells (Allen & Eroglu, 2017; Bonvento & Bolaños, 2021; Cserép et al, 2021; Pease-Raissi & Chan, 2021; Hu & Tao, 2024; Ngoc et al, 2025).

Examination of the top DEGs in NeuN^–^cells between LP-N and SP-N revealed several notable candidates associated with circadian regulation and metabolic adjustment. Among these were core circadian regulators *Dbp*, *Per2*, *Cry2*, and *Nr1d1*, all of which are closely linked to circadian rhythmicity and photoperiodic acclimatisation (Price et al, 1998; Griffin et al, 1999; Preitner et al, 2002; Ueda et al, 2005; Yoshitane et al, 2019; Patke et al, 2020). Although circadian rhythmicity is generally considered to be primarily driven by SCN neurons, the astrocyte-specific expression of the core clock gene *Cry1* has been shown to autonomously initiate and sustain complex circadian behaviour in mice, highlighting the importance of circadian gene expression in glial cells (Brancaccio et al, 2019). Interestingly, previous studies demonstrated that down-regulation of *Nr1d1* in astrocytes reduced neuronal survival in co-cultured motor neurons (Killoy et al, 2022), whereas deletion of *Nr1d1* in microglia induced a pro-inflammatory phenotype associated with impaired neuronal health (Griffin et al, 2019). Together, these findings are consistent with a potentially supportive role of the observed *Nr1d1* induction in non-neuronal cells during seasonal reprogramming.

In addition to circadian pathways, the observed transcriptional changes also pointed to alterations in metabolic regulation. Notably, we observed induction of *Acsl6*, a gene essential for enrichment of the neuroprotective omega-3 fatty acid docosahexaenoic acid in the brain. Loss of *Acsl6* has been associated with altered metabolism, impaired motor function, and increased astrogliosis and microglial activation, suggesting an important role of *Acsl6*-mediated lipid metabolism in maintaining neuronal homeostasis and neurological health (Fernandez et al, 2018). We also identified *Acss2* as a gene with increased expression in non-neuronal cells. *Acss2* is known to play an important role in metabolic regulation (El-Kurjieh et al, 2025). Interestingly, a previous study demonstrated a neuroprotective effect of *Acss2* in the context of neurodegenerative disease through maintenance of histone acetylation and regulation of neuronal gene expression, although the work focused primarily on neurons rather than glial cells (Pourshafie et al, 2026). Therefore, these findings suggest that non-neuronal cells undergo seasonal reprogramming characterised by induction of genes that may contribute to maintaining neuronal integrity during the metabolic challenge of daily torpor.

In contrast to the seasonal changes observed in non-neuronal cells, cortical neurons exhibited prominent transcriptional regulation specifically during torpor. In particular, we identified differential regulation of pathways associated with RNA metabolism and RNA catabolism. Because RNA and protein syntheses are highly energy-demanding cellular processes, these changes may reflect adjustments to the hypometabolic state. However, the functional consequences of these transcriptional changes remain unclear. Our data therefore primarily demonstrate that cortical neurons undergo pronounced regulation of RNA-associated processes during torpor. Although these transcriptional changes may be reversible, additional experiments including sampling during or immediately after arousal would be required to determine the reversibility and temporal dynamics of these RNA-associated processes.

Similar mechanisms have been described in other hibernating species. For example, in the CNS of the thirteen-lined ground squirrel (*Ictidomys tridecemlineatus*), protein synthesis is markedly suppressed during hibernation through increased phosphorylation of eukaryotic initiation factor 2α and modulation of mammalian target of rapamycin signalling (Frerichs et al, 1998). Another study in the same species demonstrated widespread, temperature-sensitive A-to-I RNA editing in the brain during torpor, predominantly targeting double-stranded RNA structures formed by repetitive elements. Editing frequencies progressively increase throughout the torpor bout and return to baseline upon arousal, reflecting a reversible and dynamic regulation of RNA metabolism. This mechanism is thought to prevent the accumulation of dsRNA that could otherwise activate maladaptive innate immune responses (Riemondy et al, 2018). Similarly, a transcriptome-wide analysis in thirteen-lined ground squirrels demonstrated dynamic regulation of RNA stability and alternative splicing during torpor–arousal cycles (Fu et al, 2021). The authors showed that changes in transcript abundance during torpor are partly explained by differences in RNA stability, highlighting the importance of post-transcriptional regulation during hypometabolism. As an additional example of post-transcriptional control, they also identified pronounced regulation of miRNA precursor transcripts during hibernation. Together, these findings suggest that regulation of RNA metabolism and protein synthesis is an evolutionarily conserved strategy to preserve neuronal integrity during hypometabolic states.

Furthermore, we observed striking differences between the transcriptional changes in the cortex and those previously reported in the hypothalamus. We reanalysed a publicly available RNA-sequencing dataset from Bao et al (2019), which compared hypothalamic gene expression between LP-N and SP-N. When examining the expression of the top DEGs from this dataset in our cortical dataset, we observed limited overlap. This limited concordance may reflect differences in experimental design. They compared only seasonal states (LP-N versus SP-N), whereas our study in addition compared the normothermia-to-hypothermia transition within the SP condition (SP-N versus SP-T). Furthermore, their dataset derives from bulk hypothalamic tissue, whereas our analysis was performed in cell type–specific cortical populations. Most importantly, these findings highlight distinct transcriptional responses in the hypothalamus and cortex during seasonal adaptation and torpor.

These findings are consistent with earlier work by Schwartz and colleagues, who performed bulk RNA sequencing of the cerebral cortex and hypothalamus across different stages of the hibernation cycle in thirteen-lined ground squirrels (*Ictidomys tridecemlineatus*). Although more than 90% of the overall mRNA repertoire was shared between the two brain regions, only ∼20% of the DEGs overlapped, indicating highly region-specific transcriptional responses during hibernation (Schwartz et al, 2013). This regional specialisation is consistent with the distinct functional roles of these brain regions. The hypothalamus acts as a central regulator of seasonal acclimatisations and torpor, whereas the cortex appears to play a more passive role. In *Phodopus sungorus*, the onset of torpor season is triggered by short-day photoperiods. Light information is transmitted via the retinohypothalamic tract to the SCN, the master circadian clock, and further processed by hypothalamic nuclei such as the paraventricular and dorsomedial hypothalamus. These structures integrate environmental cues and orchestrate pleiotropic metabolic and thermoregulatory adjustments (van Esseveldt et al, 2000; Cedernaes et al, 2019). In contrast, the cortex does not directly participate in the initiation of torpor. Our aim was therefore not to investigate the induction of torpor, but rather to understand how cortical neurons survive periods of metabolic depression and low temperature.

These findings are particularly relevant for the development of new therapeutic strategies for human diseases, as metabolic depression and altered energy availability in the central nervous system are observed in many neurological disorders (Wilson et al, 2023; Woo et al, 2024; Yuan et al, 2025). For example, in stroke, the most common cause for neurological disabilities and mortality, the supply of oxygen and nutrients is interrupted because of vessel occlusion (Hilkens et al, 2024). Neurons are known to undergo translational arrest during acute ischaemia, resembling aspects of our observations during torpor (Kleihues & Hossmann, 1971; Hu & Wieloch, 1993; DeGracia, 2017). However, after reperfusion, neurons in vulnerable brain regions often fail to restore translational capacity, ultimately leading to delayed neuronal death (Jamison et al, 2008). Moreover, dysregulated RNA metabolism is a hallmark of several neurodegenerative disorders, underscoring the importance of tightly regulated RNA homeostasis for neuronal survival (Liu et al, 2017). These observations highlight subtle but important differences between programmed and pathological energy shortage. Understanding how cortical neurons temporarily switch to an alternative mode of RNA metabolism during torpor may therefore provide valuable insights into protective mechanisms that could be leveraged for novel neuroprotective strategies.

In this study, we employed neuronal nuclei sorting followed by bulk RNA sequencing, which enabled us to distinguish between neuronal and non-neuronal populations. However, bulk sequencing inherently limits resolution, preventing the discrimination of different neuronal subtypes or glial cells. A promising next step would be to use single-nucleus RNA sequencing, which could provide a more granular view of transcriptional responses (Poulin et al, 2016; Ofengeim et al, 2017). Moreover, although we identified differential gene expression in specific pathways in neurons during torpor, the upstream mechanisms driving these changes remain unclear. Investigating epigenetic regulation, for example, by ChIP-seq to assess transcription factor binding or histone modifications, could shed light on these mechanisms (Rotem et al, 2015; Ma & Zhang, 2020). In addition, post-transcriptional regulation is likely to play an important role. In the Rickett’s big-footed bat (*Myotis ricketti*), hibernation is accompanied by dramatic changes in the brain miRNA profile (Yuan et al, 2015). Studying miRNA-mRNA networks in the Djungarian hamster may therefore provide important insights into how transcriptional and RNA metabolic changes are controlled, because the crucial role of miRNAs in regulating cellular responses in both healthy and disease contexts is well established (McNeill & Van Vactor, 2012; Gebert & MacRae, 2019; Winkler et al, 2023).

In summary, our findings reveal that cortical neurons employ reversible reprogramming of RNA metabolism to withstand metabolic depression during torpor, whereas glial cells provide long-term seasonal support. This strategy is distinct from the transcriptional programmes observed in the hypothalamus, underscoring region-specific mechanisms of survival. Elucidating these protective pathways may not only advance our understanding of torpor biology but also open new avenues for therapies aimed at enhancing neuronal resilience in human disease.

## Materials and Methods

### Animals

Djungarian hamsters (*Phodopus sungorus*) were bred and raised at the Institute of Molecular Endocrinology and Physiology at Ulm University. Until adulthood, animals were single-housed in Makrolon type III cages at Ta 20 ± 1 under LP conditions (16:8 h light:dark), with food (Altromin hamster breeding diet 7014) and water provided ad libitum. At the age of 17 ± 3 wk, five animals remained in LP and eight animals were transferred to SP (8:16 h light:dark). Initial body weight was 36.4 ± 6,8 g. After 12 ± 1 wk in SP and a body weight reduction to 25.6 ± 2 g, hamsters were implanted intraperitoneally with a radio transmitter (DSI) for continuous monitoring of body temperature. Animals were anaesthetised using isoflurane (2.5% and 1 ml min–1 for induction, 0.75–2.0% and 0.4 ml min–1 for maintenance), and analgesia was provided by carprofen (Rimadyl, 5 g kg–1 animal). Body mass, coat care, posture, and behaviour were monitored daily for seven days until recovery. Experimental and surgical procedures were approved by the local animal welfare authorities (Regional Council Tübingen, licence 1411). A total of 13 male and female animals were used. Animal euthanasia was synchronised to ZT0 and ZT4, where ZT0 corresponds to the onset of the photophase and ZT4 to 4 h after photophase onset. Five animals (3 males and 2 females) were sampled at ZT4 in LP conditions with a terminal body mass of 33.8 ± 3.4 g. Eight animals were sampled after 16 ± 2 wk in SP with a terminal body mass of 26.7 ± 1.6 g. Of the eight SP-adapted animals, four (3 males and 1 female) were culled during torpor at ZT4 (Tb 24.6 ± 1°C), and four (2 males and 2 females) were in a normothermic state on a day without torpor at the same ZT (Tb 35.3 ± 0.3°C). Animals were culled using CO2 and perfused immediately with ice-cold PBS; whole brains were dissected. For further analysis, we isolated the isocortex, comprising all six cortical layers. This comprised the entire isocortex from both hemispheres and across the whole brain. Dissection excluded other regions, including the olfactory cortex, hippocampus, thalamus, hypothalamus, and brainstem. The tissue was snap-frozen on dry ice and stored at −80°C until further processing. Transcriptome data from the hypothalamus of 12 individuals from this study have previously been published (Haugg et al, 2022).

### Isolation of neuronal nuclei

Frozen cortices, including both hemispheres from each animal, were thawed on ice. The tissue was transferred to 2 ml of EZ buffer (catalogue no. NUC101; Sigma-Aldrich) and dissociated using a glass tissue grinder. EZ buffer is a cell lysis buffer designed to gently disrupt cellular membranes while preserving intact nuclei. After a 5-min incubation on ice, the homogenate was centrifuged (500*g*, 5 min, 4°C). The pellet was washed once in 2 ml EZ buffer, followed by two washes in nuclei incubation buffer (340 mM sucrose, 2 mM MgCl_2_, 25 mM KCl, 65 mM glycerophosphate, 5% glycerol, 1 mM EDTA, 1% bovine serum albumin) (Rothammer et al, 2022).

### Flow cytometric sorting of neuronal nuclei from the cortex

Nuclei were filtered through a 30-μm mesh, followed by staining with AF647-labelled NeuN antibody (1:500, Ab190565; Abcam) and 0.25  μg ml^−1^ propidium iodide (421301; BioLegend) as a DNA counterstain. Staining was performed on ice for approximately 10–20 min. NeuN+ nuclei were sorted using a BD FACSAria III cell sorter (BD Biosciences). Sorted nuclei were collected directly into tubes pre-filled with RLT buffer and maintained at 4°C during sorting. After sorting, samples were kept on ice until all samples had been processed.

### RNA isolation

RNA was isolated by lysing samples in RLT buffer (RNeasy Mini Kit, Qiagen) supplemented with β-mercaptoethanol (1:100) and homogenised using QIAshredder columns (Qiagen). RNA was purified from the flow-through using silica membrane spin columns (RNeasy Mini Kit) according to the manufacturer’s protocol. The RNA was eluted in 30 μl RNase-free H_2_O. NanoPhotometer N60 (Implen) was used for measuring RNA concentration and verifying RNA purity. Afterwards, RNA was stored at −80°C.

### Bulk RNA sequencing

RNA-sequencing libraries were prepared using the TruSeq stranded mRNA Library Prep Kit (Illumina) according to the manufacturer’s instructions. Libraries were pooled and sequenced on a NovaSeq 6000 sequencer (Illumina) generating 50-bp paired-end reads. Reads were aligned to the Ensembl mouse reference genome (GRCm39) using STAR v.2.4 with default parameters, as previously applied to transcriptomic studies in the Djungarian hamster. Differential expression analysis was performed with DESeq2 (v.3.12), identifying DEGs as those showing ≥ twofold change with a false discovery rate–adjusted *P* < 0.05. Overlaps with annotated gene loci were counted using featureCounts v.1.5.1. Gene lists were annotated with biomaRt (v.4.0), and GSEA was conducted using clusterProfiler. Circular gene–pathway network visualisations were generated using the emapplot and cnetplot functions from the clusterProfiler package with default settings. The emapplot analysis included the 30 most significant GO terms; cnetplot visualisations were generated using the four most significantly regulated GO terms (Xu et al, 2024).

## Supplementary Material

Reviewer comments

## Data Availability

RNA-sequencing data have been deposited in the Gene Expression Omnibus repository (https://www.ncbi.nlm.nih.gov/geo/) under the accession number GSE320478. All the remaining data are included in the article or are available from the corresponding author upon reasonable request.
