# Supplementary material for: The transcriptional cortical response to daily torpor in Phodopus sungorus
Source: Life Sci Alliance. 2026 Jul 29;9(10):e202603677. doi: 10.26508/lsa.202603677 (PMC13421379; doi:10.26508/lsa.202603677)
Supplement: Reviewer comments [file LSA-2026-03677_review_history.pdf]

# The transcriptional cortical response to daily torpor in Phodopus sungorus

Lukas Bal, Marcel Woo, Nicola Rothammer, Elena Haugg, Christina Mayer, Annika Herwig, and Manuel Friese
**DOI: https://doi.org/10.26508/lsa.202603677**

*Corresponding author(s): Manuel Friese, University Medical Center Hamburg-Eppendorf*



| **Review Timeline:** | | |
|---|---|---|
| | Submission Date: | 2026-02-26 |
| | Editorial Decision: | 2026-04-07 |
| | Revision Received: | 2026-05-27 |
| | Editorial Decision: | 2026-07-03 |
| | Revision Received: | 2026-07-14 |
| | Accepted: | 2026-07-15 |

*Scientific Editor: Sarita Hebbar*


## Transaction Report:

(Note: With the exception of the correction of typographical or spelling errors that could be a source of ambiguity, letters and reports are not edited. The original formatting of letters and referee reports may not be reflected in this compilation.)

April 7, 2026

Re: Life Science Alliance manuscript #LSA-2026-03677

Prof. Manuel Alexander Friese
University Medical Center Hamburg-Eppendorf
Institute of Neuroimmunology and Multiple Sclerosis
Falkenried 94
Hamburg D-20251
Germany

Dear Dr. Friese,

Thank you for submitting your manuscript entitled "The transcriptional cortical adaptations to daily torpor in Phodopus sungorus" to Life Science Alliance. Your manuscript was evaluated by three reviewers whose comments are included below. As you will note, the three reviewers find your work of value, and Reviewers 1 and 3 comment positively on the additional focus on gene expression changes in cortical non-neuronal cells. That said, all the reviewers have also highlighted areas of the manuscript that need further attention. We invite you to submit a revised manuscript addressing all of the three reviewers' concerns.
We agree with all three reviewers that you must (1) contextualise your work within the field (in terms of existing published information), (2) expand on your discussion of these findings, and (3) improve the clarity and completeness of the manuscript. We also encourage you to pay attention to the specific use of certain terminology and nomenclature as indicated by the reviewers.

I would be happy to discuss the revision in more detail via email or phone/videoconferencing. Please let me know which option you prefer, if any.

To upload the revised version of your manuscript, please log in to your account: https://lsa.msubmit.net/cgi-bin/main.plex

You will be guided to complete the submission of your revised manuscript and to fill in all necessary information. Please get in touch in case you do not know or remember your login name.

The typical timeframe for revisions is three months. Please note that papers are generally considered through only one revision cycle, so strong support from the referees on the revised version is needed for acceptance.

While you are revising your manuscript, please also attend to the below editorial points to help expedite the publication of your manuscript. Please direct any editorial questions to the journal office. When submitting the revision, please include a letter addressing the reviewers' comments point by point.

Thank you for this interesting contribution to Life Science Alliance. We hope that the comments below will prove constructive as your work progresses, and we are looking forward to receiving your revised manuscript.

Sincerely,

Sarita Hebbar, PhD
Scientific Editor
Life Science Alliance
http://www.lsajournal.org

---------------------------------------------------------------------------

A. THESE ITEMS ARE REQUIRED FOR REVISIONS

-- A letter addressing the reviewers' comments point by point.

-- An editable version of the final text (.DOC or .DOCX) is needed for copyediting (no PDFs).

-- High-resolution figure, supplementary figure and video files uploaded as individual files: See our detailed guidelines for preparing your production-ready images, https://www.life-science-alliance.org/authors

-- Summary blurb (enter in submission system): A short text summarizing in a single sentence the study (max. 200 characters including spaces). This text is used in conjunction with the titles of papers, hence should be informative and complementary to

the title and running title. It should describe the context and significance of the findings for a general readership; it should be written in the present tense and refer to the work in the third person. Author names should not be mentioned.

-- By submitting a revision, you attest that you are aware of our payment policies found here: https://www.life-science-alliance.org/copyright-license-fee

B. MANUSCRIPT ORGANIZATION AND FORMATTING:

Full guidelines are available on our Instructions for Authors page, https://www.life-science-alliance.org/authors

We encourage our authors to provide original source data, particularly uncropped/-processed electrophoretic blots and spreadsheets for the main figures of the manuscript. If you would like to add source data, we would welcome one PDF/Excel-file per figure for this information. These files will be linked as supplementary "Source Data" files.

***IMPORTANT: It is Life Science Alliance policy that if requested, original data images must be made available. Failure to provide original images upon request will result in unavoidable delays in publication. Please ensure that you have access to all original microscopy and blot data images before submitting your revision.***

---------------------------------------------------------------------------

Reviewer #1 (Comments to the Authors (Required)):

The new data presented in this work compares transcriptome changes in neurons and non-neuronal cells of the "cortex" from three physiological states of Phodopus; 1) long-day normothermic, 2) short day normothermic and 3) short day hypothermic (torpid) individuals. It also re-evaluates a published dataset from RNA-seq of the hypothalamus to compare to the new cortex data.

The main strength of this work is that it is the first report of gene expression dynamics in cortical neuronal vs. non-neuronal cells in a hibernator. The data are convincing that the neuronal and non-neuronal cells are distinct and respond differently to seasonal preparation for hibernation (found in non-neuronal cells but not neuronal cells) and temperature (in both, with distinct functional enrichments).

The main weakness of this work is that it fails to adequately represent and build on related results found in prior literature.

Specific questions and concerns:
How exactly was cortex dissected for this work? (Methods say only cortices removed immediately?) Is it literally the 6 cell layers of cerebral cortex? From which brain area?

The abbreviations for the distinct physiological states seem unnecessarily confusing - LPNT, SPNT and SPHT for long photoperiod, (LP) short photoperiod (SP) normothermic (NT) and hypothermic (HT). Because the goal is to contrast transcriptomes among 3 groups, normothermic in short vs long photoperiod and torpid in short photoperiod, the T is easily confused for torpid, why not use a simpler nomenclature like LN, SN and ST? Or LP-N, SP-N and SP-T? It is particularly crucial to avoid the term "hypothermic "when referring to natural torpor which is not hypothermia.

Introduction, Results and Discussion: The similarities and differences between these findings and those of two earlier papers (PMID: 23526982, PMID: 33536943) that have documented gene expression differences in hypothalamus and "cortex" are not adequately described or discussed. Science builds on prior knowledge; changes in RNA metabolism, and differences among brain regions in torpid hibernators vs normothermic hibernators as well as seasonal differences have all been described elsewhere.

Introduction: The correct reference for the two-switch model of hibernation is PMID: 17536023.

Introduction and Discussion: Hypophosphorylation of eIF2alpha has nothing to do with transcription, it is curious that this paper about translation elongation is highlighted instead of the far more relevant papers noted above.

The term "adaptation" is misused throughout. This word indicates a change that improves fitness. There is no evidence to support a conclusion that any of the observed transcriptomic changes are adaptive. Differences do not need to be adaptive to exist.

Methods and Figures (4 and 5): Network analyses are not adequately described or referenced.

Reviewer #2 (Comments to the Authors (Required)):

The manuscript presents a transcriptomic analysis of genes in the cerebral cortex of the Djungarian hamster during torpor and non-torpor conditions. The authors report that transcriptional changes in the cortex appear to be primarily associated with hypothermia rather than photoperiod, which contrasts with previous observations in the hypothalamus where photoperiod has a strong influence on gene expression. The study addresses an interesting question and contributes to the understanding of brain region-specific transcriptional responses during torpor.

Before commenting further, I would like to note that my expertise is mainly in physiology of the autonomic nervous system rather than molecular biology. Therefore, I am not in a position to evaluate in detail the bioinformatic pipelines, gene annotation procedures, or the specific databases used for the transcriptomic analysis.

However, I would like to raise a few points that could improve the clarity and completeness of the manuscript.

First, the sex of the animals used in the study is not specified. This information is important and should be clearly reported. Sex differences may influence gene expression patterns, and in females cyclic hormonal variations could potentially affect transcriptional profiles. Therefore, the authors should indicate whether animals were male, female, or both, and if applicable clarify how this variable was controlled.

Second, the introduction aims to provide a general overview of hibernation and torpor. However, several statements appear to describe very specifically the Djungarian hamster model used in the study. While these characteristics are correct for this species, they are not representative of many other species that exhibit daily torpor or seasonal hibernation. For example, many heterothermic species do not undergo seasonal coat color changes and may not reach such low body temperatures. I would suggest that the authors either (1) broaden the description to more accurately reflect the diversity of hibernating and torpor-expressing species, or (2) clearly state that the described features refer specifically to the Djungarian hamster model.

Addressing these points would help improve the general clarity of the manuscript and avoid potential overgeneralizations.

Reviewer #3 (Comments to the Authors (Required)):

The paper assesses changes in cortical cells during torpor in Djungarian hamsters. The study finds that non neuronal cells show seasonal adaptation within the cortex, whereas neuronal cells are more susceptible to metabolic changes than seasonal changes. I am personally pleased to see some focus on non-neuronal cells within the field.

The methodology can be improved by adding some details and clarification. I encourage the authors to add information as it would be very helpful if another lab wants to perform the same experiments or analyses. If published protocols have been followed that provide the necessary details to reproduce the experiments, they should be cited. Below are some of the questions that I would have if I were trying to reproduce the experiments:
• What is the sex of the animals?
• If animals were culled with $CO_2$, did they get perfused post-mortem?
• Were both hemispheres used for analyses? Was the homogenisation with a glass douncer performed on ice?
• Were any enzymes added during tissue dissociation?
• How samples were handled/transferred from the flow cytometry sorting to the next RNA isolation step? Sorted into tubes? Were they kept on ice? For how long? Were cells diluted?
• Was the density of the cells measured?
• Were any controls used (e.g. FMO)? How long and at what temperature were the samples incubated with the antibodies?
• Was the viability of the cells measured?
• How were RNA quality and integrity assessed?
• Fig1. The flow cytometry graph shows propidium iodide, but it is not mentioned in the methods. Please add to the methods. Where the cell sorted in 3 groups: Neurons, non-neurons, non-viable (PI)? Was Hoechst33582 used (please add manufacturer)?
• Was RNA quantified? Was cDNA generated?
• Were data normalized? What was baseline used for the 2-fold change?

Regarding the results section I was wondering if the Gene Ontology (GO) enrichment analysis on DEGs performed on NeuN- population, to confirm isolation of non neuronal cells? If so, that could be added.

Overall, I think a revision of the discussion would strongly benefit the paper, by detailing the differences found between SPHT vs STNT and hypothalamus and cortex that would support the aim of the paper "to understand how cortical neurons survive periods of metabolic depression and low temperature"; as the discussion is currently written, I am unsure how increasing RNA catabolic pathways would preserve CNS integrity during torpor (preventing accumulation of toxic components? Maintaining cellular homeostasis?). I am also unsure what is the "alternative mode of RNA metabolism during torpor", is it the increase in the RNA catabolic pathways?

There are sections that are quite abstract or require clarifications. Some examples below.

"This finding is consistent with the supportive functions of glial cells (Allen and Eroglu, 2017; Bonvento and Bolaños, 2021; Cserép et al., 2021; Hu and Tao, 2024; Ngoc et al., 2025; Pease-Raissi and Chan, 2021) and suggests that non-neuronal cells contribute to cortical metabolic adaptations during seasonal reprogramming."

It would be helpful to be a little more specific on what findings are consistent with supportive functions of glia, and state what supportive functions (as there are many). Are there some genes that stand out related to these supportive functions? I also perceive the second part of the sentence as quite vague; it would be more helpful to state how and what cortical metabolic adaptations (do metabolic adaptations refer to torpor in general ?). In general, I think it would be helpful for the reader to have more context; I encourage the authors to select the genes that shows dramatic changes (likely the top 20genes in the graphs), explain briefly what each gene does (or group of genes) and put it in the context of seasonal changes, metabolism and/or torpor. Why would an increase or decrease of that selected gene make sense (is beneficial) for torpor/seasonal adaptations? I believe that would make the discussion more accessible to readers and provide some contextualized knowledge that could trigger new ideas and area of investigation. A similar approach should be taken for the neuronal cells section as well.

"For example, in the central nervous system of the [please add Thirteen-lined] ground squirrel (Spermophilus tridecemlineatus), protein synthesis is markedly suppressed during hibernation through increased phosphorylation of eukaryotic initiation factor 2α and modulation of mammalian target of rapamycin (mTOR) signalling (Frerichs et al., 1998). Another study in hibernating [please delete hibernating] ground squirrels demonstrated widespread, temperature-sensitive A-to-I RNA editing in the brain during torpor, predominantly targeting double-stranded RNA structures formed by repetitive elements [ add Citation]. Editing frequencies progressively increase throughout the torpor bout and return to baseline upon arousal, reflecting a reversible and dynamic regulation of RNA metabolism. This mechanism is thought to prevent the accumulation of dsRNA that could otherwise activate maladaptive innate immune responses (Riemondy et al., 2018)."

Sometimes it can get confusing when writing about hibernating ground squirrel, readers may interpret that as ground squirrels during hibernation/torpor bout or obligatory hibernators, I suggest for clarity to just say the ground squirrel's species. I believe the official/most recent nomenclature for the 13-lined squirrel is Ictidomys tridecemlineatus.

-Also, the paper implies that RNA catabolism is reactivated upon arousal, wouldn't this require samples collected during arousal or just after arousal?

-Finally, I strongly encourage the authors to incorporate into the discussion the paper below, which also show different profile between cortex and hypothalamus:
Schwartz C, Hampton M, Andrews MT (2013) Seasonal and Regional Differences in Gene Expression in the Brain of a Hibernating Mammal. PLOS ONE 8(3): e58427. https://doi.org/10.1371/journal.pone.0058427

'Referee Cross-Comments'

**POINT-TO-POINT RESPONSE TO REFEREES – #LSA-2026-03677**

The Reviewers' comments are in *blue italics* and our responses are listed below each statement. Changes in the manuscript have been highlighted in red.

**Reviewer #1**

*The new data presented in this work compares transcriptome changes in neurons and non-neuronal cells of the "cortex" from three physiological states of Phodopus; 1) long-day normothermic, 2) short day normothermic and 3) short day hypothermic (torpid) individuals. It also re-evaluates a published dataset from RNA-seq of the hypothalamus to compare to the new cortex data. The main strength of this work is that it is the first report of gene expression dynamics in cortical neuronal vs. non-neuronal cells in a hibernator. The data are convincing that the neuronal and non-neuronal cells are distinct and respond differently to seasonal preparation for hibernation (found in non-neuronal cells but not neuronal cells) and temperature (in both, with distinct functional enrichments). The main weakness of this work is that it fails to adequately represent and build on related results found in prior literature. Specific questions and concerns:*

**Response:** We thank the Reviewer for the overall positive assessment of our manuscript and the constructive comments and suggestions that have led to a further improved quality of the manuscript.

*1. How exactly was cortex dissected for this work? (Methods say only cortices removed immediately?) Is it literally the 6 cell layers of cerebral cortex? From which brain area?*

**Response:** As requested, we have clarified the description of the cortical dissection in the Methods section.

*2. The abbreviations for the distinct physiological states seem unnecessarily confusing - LPNT, SPNT and SPHT for long photoperiod, (LP) short photoperiod (SP) normothermic (NT) and hypothermic (HT). Because the goal is to contrast transcriptomes among 3 groups, normothermic in short vs long photoperiod and torpid in short photoperiod, the T is easily confused for torpid, why not use a simpler nomenclature like LN, SN and ST? Or LP-N, SP-N and SP-T? It is particularly crucial to avoid the term "hypothermic "when referring to natural torpor which is not hypothermia.*

**Response:** We agree with the Reviewer and have changed the abbreviations to LP-N, SP-N and SP-T throughout the manuscript.

*3. Introduction, Results and Discussion: The similarities and differences between these findings and those of two earlier papers (PMID: 23526982, PMID: 33536943) that have documented gene expression differences in hypothalamus and "cortex" are not adequately described or discussed. Science builds on prior knowledge; changes in RNA metabolism, and differences among brain regions in torpid hibernators vs normothermic hibernators as well as seasonal differences have all been described elsewhere.*

**Response:** We thank the Reviewer for highlighting the relevant prior studies. We have now incorporated these findings into the Introduction and Discussion sections. The study by Fu and colleagues from 2021 was already mentioned in the Discussion of the previous version, but we have now further described their findings.

*4. Introduction: The correct reference for the two-switch model of hibernation is PMID: 17536023.*

**Response:** We thank the Reviewer for pointing this out and for providing the correct reference. We have included the citation accordingly in the revised manuscript.

*5. Introduction and Discussion: Hypophosphorylation of eIF2alpha has nothing to do with transcription, it is curious that this paper about translation elongation is highlighted instead of the far more relevant papers noted above.*

**Response:** As suggested, we have incorporated the indicated studies into the Introduction and Discussion sections to provide a more comprehensive context. Although transcriptional and translational processes are distinct, we consider the cited study on translational regulation relevant within the broader context of coordinated downregulation of gene expression and protein biosynthesis. Therefore, we have added the recommended references, but have retained this citation.

*6. The term "adaptation" is misused throughout. This word indicates a change that improves fitness. There is no evidence to support a conclusion that any of the observed transcriptomic changes are adaptive. Differences do not need to be adaptive to exist.*

**Response:** We thank the Reviewer for this important comment. We have revised the manuscript and replaced the term “adaptation” with “acclimatisation” or “adjustments”.

*7. Methods and Figures (4 and 5): Network analyses are not adequately described or referenced.*

**Response:** We used the emapplot and cnetplot functions with default settings from the clusterProfiler package. The 30 most significant GO terms were used for the emapplot and the 4 most significantly regulated GO terms were used for the cnetplots. This has now been specified in the methods section.

**Reviewer #2:**

*The manuscript presents a transcriptomic analysis of genes in the cerebral cortex of the Djungarian hamster during torpor and non-torpor conditions. The authors report that transcriptional changes in the cortex appear to be primarily associated with hypothermia rather than photoperiod, which contrasts with previous observations in the hypothalamus where photoperiod has a strong influence on gene expression. The study addresses an interesting question and contributes to the understanding of brain region-specific transcriptional responses during torpor. Before commenting further, I would like to note that my expertise is mainly in physiology of the autonomic nervous system rather than molecular biology. Therefore, I am not in a position to evaluate in detail the bioinformatic pipelines, gene annotation procedures, or the specific databases used for the transcriptomic analysis. However, I would like to raise a few points that could improve the clarity and completeness of the manuscript.*

**Response:** We thank the Reviewer for the positive comments.

*1. First, the sex of the animals used in the study is not specified. This information is important and should be clearly reported. Sex differences may influence gene expression patterns, and in females cyclic hormonal variations could potentially affect transcriptional profiles. Therefore, the authors should indicate whether animals were male, female, or both, and if applicable clarify how this variable was controlled.*

**Response:** Both male and female animals were used in this study, and this information has now been added to the Methods section. SP animals are reproductively quiescent; gonadal activity and circulating sex steroids are markedly reduced in both sexes, and females are acyclic. Therefore, we do not expect substantial sex-related effects within the SP groups. Moreover, we did not observe significant transcriptional differences between SP-N and LP-N neurons despite their opposing reproductive states, suggesting that sex distribution was unlikely to be a major confounding factor in our analyses.

*2. Second, the introduction aims to provide a general overview of hibernation and torpor. However, several statements appear to describe very specifically the Djungarian hamster model used in the study. While these characteristics are correct for this species, they are not representative of many other species that exhibit daily torpor or seasonal hibernation. For example, many heterothermic species do not undergo seasonal coat color changes and may not reach such low body temperatures. I would suggest that the authors either (1) broaden the description to more accurately reflect the diversity of hibernating and torpor-expressing species, or (2) clearly state that the described features refer specifically to the Djungarian hamster model. Addressing these points would help improve the general clarity of the manuscript and avoid potential overgeneralizations.*

**Response:** We thank the Reviewer for this suggestion, which has improved the clarity and precision of the manuscript. We have now included a broader introduction to the topic of torpor in the first part of the Introduction and subsequently stated more clearly that the remainder of the Introduction focuses on the Djungarian hamster.

**Reviewer #3:**

*The paper assesses changes in cortical cells during torpor in Djungarian hamsters. The study finds that non neuronal cells show seasonal adaptation within the cortex, whereas neuronal cells are more susceptible to metabolic changes than seasonal changes. I am personally pleased to see some focus on non-neuronal cells within the field.*

*1. The methodology can be improved by adding some details and clarification. I encourage the authors to add information as it would be very helpful if another lab wants to perform the same experiments or analyses. If published protocols have been followed that provide the necessary details to reproduce the experiments, they should be cited. Below are some of the questions that I would have if I were trying to reproduce the experiments:*

1. *What is the sex of the animals?*
2. *If animals were culled with CO2, did they get perfused post-mortem?*
3. *Were both hemispheres used for analyses? Was the homogenisation with a glass douncer performed on ice?*
4. *Were any enzymes added during tissue dissociation?*
5. *How samples were handled/transferred from the flow cytometry sorting to the next RNA isolation step? Sorted into tubes? Were they kept on ice? For how long? Were cells diluted?*
6. *Was the density of the cells measured?*
7. *Were any controls used (e.g. FMO)? How long and at what temperature were the samples incubated with the antibodies?*
8. *Was the viability of the cells measured?*
9. *How were RNA quality and integrity assessed?*
10. *Fig1. The flow cytometry graph shows propidium iodide, but it is not mentioned in the methods. Please add to the methods. Where the cell sorted in 3 groups: Neurons, non-neurons, non-viable (PI)? Was Hoechst33582 used (please add manufacturer)?*
11. *Was RNA quantified? Was cDNA generated?*
12. *Were data normalized? What was baseline used for the 2-fold change?*

**Response:** We thank the Reviewer for the detailed comments and suggestions, which helped us to further improve the quality and clarity of the Methods section and thereby facilitate reproducibility for other researchers. All corresponding information has now been added to the revised manuscript. We also included a citation, where the protocol has been previously used.

Here we provide a detailed response to each aspect raised:

1. We have included this information in the method section.
2. Indeed, the animals were perfused post-mortem, immediately after being culled with CO2, followed by rapid dissection of the whole brain and separation of the cortices. To improve clarity, we have revised the corresponding section in the Methods accordingly.
3. Both hemispheres were used for all analyses. In response to the Reviewer's helpful comment, we revised the Methods section to indicate this more clearly. As described in the manuscript, tissue dissociation was performed using a glass tissue grinder.
4. No additional enzymes were used during tissue dissociation. Instead, tissue homogenization was performed using EZ buffer, as described in the manuscript. EZ buffer is a cell lysis buffer designed to gently disrupt cellular membranes while preserving intact nuclei. To further clarify the procedure, we have now added this information to the revised Methods section.

5. Nuclei were sorted directly into collection tubes pre-filled with RLT buffer. During sorting, collection tubes were maintained at 4 °C within the sorter. After sorting, samples were kept on ice while the remaining samples were processed. RNA isolation was performed immediately after completion of the sorting procedure.
6. The cell density was not measured before nucleus sorting.
7. Incubation with antibodies was performed on ice for 10 minutes. A fluorescence minus one control was not included because flow cytometric neuronal nucleus sorting is a well-established method in our hands and the scientific community. Furthermore, our protocol led to clearly separated NeuN+ and NeuN– populations (Fig. 1B). Direct viability of cells was not assessed. However, PI staining allowed discrimination between intact nuclei and debris or damaged nuclear material during flow cytometric sorting.
8. We used the NanoPhotometer N60 (Implen) to measure RNA concentration and assess RNA purity.
9. This was an error in the Methods section. We did not use Hoechst, but instead used PI staining. PI was applied to distinguish intact nuclei from debris. Subsequently, NeuN positivity was used to discriminate neuronal from non-neuronal nuclei. We have corrected this in the revised manuscript.
10. As described in the Methods section, libraries were prepared using the TruSeq Stranded mRNA Library Prep Kit (Illumina). This protocol inherently includes cDNA synthesis during library preparation. Prior to library preparation, RNA concentration was measured as an additional quality control step, as described in point 9.
11. We assume that this comment refers, among others, to Fig. 2A–C. Here, the log2 fold change always refers to the expression difference between the respective comparison groups, for example, increased expression in the NeuN-positive fraction compared with the NeuN-negative fraction.

*2. Regarding the results section I was wondering if the Gene Ontology (GO) enrichment analysis on DEGs performed on NeuN- population, to confirm isolation of non neuronal cells? If so, that could be added.*

**Response:** We agree with the review that this would be a valuable analysis to validate the isolation of neuronal nuclei. We therefore performed a gene set enrichment analysis of independent neuronal, astrocyte, and oligodendrocyte transcriptional signatures in NeuN+ nuclei vs NeuN– nuclei. While we identified a strong positive enrichment of the neuronal signature, we identified a strong negative enrichment of the astrocyte and oligodendrocyte signatures (= enrichment in NeuN– fraction). These further underline the methodological validity to separate NeuN+ and NeuN– nuclei with our method. This new enrichment plot is provided in the new **Supplementary Figure 1A.**

*3. Overall, I think a revision of the discussion would strongly benefit the paper, by detailing the differences found between SPHT vs STNT and hypothalamus and cortex that would support the aim of the paper "to understand how cortical neurons survive periods of metabolic depression and low temperature"; as the discussion is currently written, I am unsure how increasing RNA catabolic pathways would preserve CNS integrity during torpor (preventing accumulation of toxic components? Maintaining cellular homeostasis?). I am also unsure what is the "alternative mode of RNA metabolism during torpor", is it the increase in the RNA catabolic pathways?*

**Response:** We thank the Reviewer for this thoughtful comment. We agree that our initial Discussion may have overstated the functional interpretation of the observed RNA-associated pathways, since our study is primarily transcriptomic in nature. We therefore revised the

Discussion to adopt a more cautious interpretation, clarifying that we observed differential regulation of RNA metabolism, RNA processing, and RNA catabolism pathways during hypothermia in cortical neurons. In addition, we removed the wording "alternative mode of RNA metabolism."

*4. "This finding is consistent with the supportive functions of glial cells (Allen and Eroglu, 2017; Bonvento and Bolaños, 2021; Cserép et al., 2021; Hu and Tao, 2024; Ngoc et al., 2025; Pease-Raissi and Chan, 2021) and suggests that non-neuronal cells contribute to cortical metabolic adaptations during seasonal reprogramming."*
*It would be helpful to be a little more specific on what findings are consistent with supportive functions of glia, and state what supportive functions (as there are many). Are there some genes that stand out related to these supportive functions? I also perceive the second part of the sentence as quite vague; it would be more helpful to state how and what cortical metabolic adaptations (do metabolic adaptations refer to torpor in general ?). In general, I think it would be helpful for the reader to have more context; I encourage the authors to select the genes that shows dramatic changes (likely the top 20genes in the graphs), explain briefly what each gene does (or group of genes) and put it in the context of seasonal changes, metabolism and/or torpor. Why would an increase or decrease of that selected gene make sense (is beneficial) for torpor/seasonal adaptations? I believe that would make the discussion more accessible to readers and provide some contextualized knowledge that could trigger new ideas and area of investigation. A similar approach should be taken for the neuronal cells section as well.*

**Response:** Following this advice, we substantially revised parts of the Discussion to provide a more detailed and contextualized interpretation of the transcriptional changes observed in the non-neuronal fraction. In particular, we now discuss several of the most prominently regulated genes identified by the RNA-seq analysis. We believe these additions improve the clarity and biological interpretation of our findings.

*5. "For example, in the central nervous system of the [please add Thirteen-lined] ground squirrel (Spermophilus tridecemlineatus), protein synthesis is markedly (…) This mechanism is thought to prevent the accumulation of dsRNA that could otherwise activate maladaptive innate immune responses (Riemondy et al., 2018)."*
*Sometimes it can get confusing when writing about hibernating ground squirrel, readers may interpret that as ground squirrels during hibernation/torpor bout or obligatory hibernators, I suggest for clarity to just say the ground squirrel's species. I believe the official/most recent nomenclature for the 13-lined squirrel is Ictidomys tridecemlineatus.*

**Response:** We now consistently specify the species throughout the manuscript.

*6. Also, the paper implies that RNA catabolism is reactivated upon arousal, wouldn't this require samples collected during arousal or just after arousal?*

**Response:** We agree on the evaluation and included this remark in our Discussion.

*7. Finally, I strongly encourage the authors to incorporate into the discussion the paper below, which also show different profile between cortex and hypothalamus:*
*Schwartz C, (…) 2013; https://doi.org/10.1371/journal.pone.0058427*

**Response:** Similarly, Reviewer 1 also recommended inclusion of this study, and we agree that this previous work provides valuable context for interpreting our findings. We have therefore

incorporated the study into both the Introduction and the Discussion to provide a broader overview of regional transcriptional adaptations in the hibernating brain.

July 3, 2026

RE: Life Science Alliance Manuscript #LSA-2026-03677R

Prof. Manuel Alexander Friese
University Medical Center Hamburg-Eppendorf
Institute of Neuroimmunology and Multiple Sclerosis
Falkenried 94
Hamburg D-20251
Germany

Dear Dr. Friese,

Thank you for submitting your revised manuscript entitled "The transcriptional cortical response to daily torpor in Phodopus sungorus". We apologise for the delay in communication a decision due to an issue with reviewer availability.

Now your revised manuscript has been evaluated by all the original reviewers whose comments are appended below. As you will read, the reviewers are consistent that your manuscript has addressed many of their previous concerns and is now improved. That said, Reviewers 1 and 3 have noted few pending concerns. We agree that you must resolve the points related to terminology, methodology details, and citations. Further we request you to check language throughout to ensure accuracy and to change the heading of results related to differential gene expression (Reviewer 1, point 3). These are relatively minor edits that should not take much time.

In line with the reviewers' evaluation, we would be happy to publish your paper in Life Science Alliance pending resolution of the pending concerns of Reviewer 1 ad 3, and final revisions necessary to meet our formatting guidelines. Please provide a revised manuscript with the changes highlighted.

MANUSCRIPT ORGANIZATION AND FORMATTING:

To avoid unnecessary delays in the acceptance and publication of your paper, please read the following information carefully. Full guidelines are available on our Instructions for Authors page, https://www.life-science-alliance.org/authors

**Submission of a paper that does not conform to Life Science Alliance guidelines will delay the acceptance of your manuscript.**
-Figure S1 has only one panel; therefore, please remove the label A from the current figure and its legend.
-Please add the X and Bluesky handles of your host institute/organization, as well as your own, and/or one of the authors, in our system.
-Please consult our manuscript preparation guidelines https://www.life-science-alliance.org/manuscript-prep and make sure your manuscript sections are in the correct order.
-Please move your main and supplementary figure legends after the references section.
-Please add a Conflict of Interest statement to your main manuscript text
-Please be sure that the authorship listing and order is correct

We encourage our authors to provide original source data, particularly uncropped/-processed electrophoretic blots and spreadsheets for the main figures of the manuscript. If you would like to add source data, we would welcome one PDF/Excel-file per figure for this information. These files will be linked as supplementary "Source Data" files.

It is Life Science Alliance policy that if requested, original data images must be made available to the editors. Failure to provide original images upon request will result in unavoidable delays in publication. Please ensure that you have access to all original data images prior to final submission.

If you are planning a press release on your work, please inform us immediately to allow informing our production team and scheduling a release date.

We welcome submissions of potential cover images for the issue of LSA in which your work would appear. If you have high quality images associated with this work, please feel free to email these, with a caption, to the journal office.

LSA encourages authors to provide a 30-60 second video where the study is briefly explained. These videos will be appear embedded with the manuscript online at Life Science Alliance, and on social media to promote the published paper and authors (for examples, see https://docs.google.com/document/d/1-UWCfbE4pGcDdcgzcmiuJl2XMBJnxKYeqRvLLrLSo8s/edit?usp=sharing). Corresponding or first-authors are welcome to submit the video. Please submit only one video per manuscript.

The video can be emailed to contact@life-science-alliance.org

FINAL FILES:

To upload the final version of your manuscript, please log in to your account: https://lsa.msubmit.net/cgi-bin/main.plex
You will be guided to complete the submission of your revised manuscript and to fill in all necessary information. Please get in touch in case you do not know or remember your login name.

The following items are required for acceptance.

-- An editable version of the final text (.DOC or .DOCX) is needed for copyediting (no PDFs).

-- High-resolution figure, supplementary figure and video files uploaded as individual files: See our detailed guidelines for preparing your production-ready images, https://www.life-science-alliance.org/authors

-- Summary blurb (enter in submission system): A short text summarizing in a single sentence the study (max. 200 characters including spaces). This text is used in conjunction with the titles of papers, hence should be informative and complementary to the title. It should describe the context and significance of the findings for a general readership; it should be written in the present tense and refer to the work in the third person. Author names should not be mentioned.

**The license to publish form must be signed before your manuscript can be sent to production. A link to the license to publish form will be available to the corresponding author only. Please take a moment to check your funder requirements.**

**Reviews, decision letters, and point-by-point responses associated with peer-review at Life Science Alliance will be published, alongside the manuscript. If you do want to opt out of having the reviewer reports and your point-by-point responses displayed, please let us know immediately.**

Thank you for your attention to these final processing requirements. Please revise and format the manuscript and upload materials as soon as you are able.

Thank you for this interesting contribution to the literature. We look forward to publishing your paper in Life Science Alliance.

Sincerely,

Sarita Hebbar, PhD
Scientific Editor
Life Science Alliance
http://www.lsajournal.org

------------------------------------------------------------------------------
Reviewer #1 (Comments to the Authors (Required)):

As noted initially, this study is the first to differentiate the response of neurons and non-neurons to seasonal and temperature effects of torpor utilization. The data are convincing and provide an important addition to the field. The authors have largely alleviated my concerns in this revision, however, I still have some significant but easily-addressable concerns about the presentation, specifically:
1) It is still unclear exactly what tissue was used for RNA isolation. The image in Figure 1 suggests the RNA came from a dashed-line enclosed cross-section of the hamster brain(although the dashed lines are not mentioned in the legend). Is the area used from a single cross section (if so which section), or from the entire brain? How much tissue was used for RNA isolation? Please be specific, the goal of reporting methods is that an interested reader could repeat exactly what was done and get the same results, which would not be possible with these methods as currently written.
2) The Fu and Schwartz papers describing similar findings in other hibernating species are both now included in the discussion, although the Fu paper is still not cited in the reference section.
3) The authors have measured differential gene expression, not differential regulation or transcriptional regulation, yet these terms are used interchangeably throughout. Examples: the section heading "Neurons upregulate RNA catabolism during torpor" should be "Genes related to RNA catabolism increased during torpor" to be accurate. This is a problem throughout, but particularly unacceptable in results and figure legends when specifically describing measurements of differential gene expression.
4) The term hypothermia should not be used in the context of torpor; it is defined by a constellation of physiological features distinct from those of natural torpor.
5) Finally, the new ref to the work of Bao et al. and the lack of overlap between the findings of this work and that of Bao in Figure 6 is, but should not be, ignored in the Discussion.

Reviewer #2 (Comments to the Authors (Required)):

the authors have made appropriate changes and I would recommend acceptance.

Reviewer #3 (Comments to the Authors (Required)):

The manuscript has improved significantly. I have enjoyed the introduction, and I am looking forward to passing it along to my students. I really appreciate the authors' effort in contextualizing their findings, the discussion and the conclusions are much more clear. Thank you!

I only have a few comments
Line 57: specify that the two switch model is for circannual hibernators
Sentence extending from line 73 to Line 75 need citation
Line 152: please add NeuN antibody company and catalogue number

**POINT-TO-POINT RESPONSE TO REFEREES – #LSA-2026-03677R**

The reviewers' comments are in *blue italics* and our responses are listed below each statement. Changes in the manuscript have been highlighted in red.

**Reviewer comments:**

**REVIEWER #1**

*As noted initially, this study is the first to differentiate the response of neurons and non-neurons to seasonal and temperature effects of torpor utilization. The data are convincing and provide an important addition to the field. The authors have largely alleviated my concerns in this revision, however, I still have some significant but easily-addressable concerns about the presentation.*

*1) It is still unclear exactly what tissue was used for RNA isolation. The image in Figure 1 suggests the RNA came from a dashed-line enclosed cross-section of the hamster brain(although the dashed lines are not mentioned in the legend). Is the area used from a single cross section (if so which section), or from the entire brain? How much tissue was used for RNA isolation? Please be specific, the goal of reporting methods is that an interested reader could repeat exactly what was done and get the same results, which would not be possible with these methods as currently written.*

**Response:** In this experiment, the entire isocortex across the whole brain was used. We have clarified this in the Methods section and revised the figure legend accordingly.

*2) The Fu and Schwartz papers describing similar findings in other hibernating species are both now included in the discussion, although the Fu paper is still not cited in the reference section.*

**Response:** We thank the reviewer for pointing out this mistake. The paper by Fu et al. has now been added to the reference section.

*3) The authors have measured differential gene expression, not differential regulation or transcriptional regulation, yet these terms are used interchangeably throughout. Examples: the section heading "Neurons upregulate RNA catabolism during torpor" should be "Genes related to RNA catabolism increased during torpor" to be accurate. This is a problem throughout, but particularly unacceptable in results and figure legends when specifically describing measurements of differential gene expression.*

**Response:** We agree with the reviewer that precise wording is essential to accurately describe our findings. We have revised the wording throughout the manuscript accordingly.

*4) The term hypothermia should not be used in the context of torpor; it is defined by a constellation of physiological features distinct from those of natural torpor.*

**Response:** We revised the manuscript and adapted the wording accordingly to improve precision. Nevertheless, to distinguish between the SP-N and SP-T groups, we have retained the term "normothermia-to-hypothermia transition" in some instances, as daily torpor in the two-switch model is characterized by a decrease in body temperature.

*5) Finally, the new ref to the work of Bao et al. and the lack of overlap between the findings of this work and that of Bao in Figure 6 is, but should not be, ignored in the Discussion.*

**Response:** We agree that further discussion of the limited overlap between our findings and those of Bao et al. is warranted. We have now included a discussion of this point in the revised manuscript.

## REVIEWER #2

*The authors have made appropriate changes and I would recommend acceptance.*

**Response:** We thank the reviewer for the previous comments and the positive response.

## REVIEWER #3

*The manuscript has improved significantly. I have enjoyed the introduction, and I am looking forward to passing it along to my students. I really appreciate the authors' effort in contextualizing their findings, the discussion and the conclusions are much more clear. Thank you!*

**Response:** We thank the reviewer for the constructive comments and the positive evaluation of our manuscript.

1) I only have a few comments; Line 57: specify that the two switch model is for circannual hibernators; Sentence extending from line 73 to Line 75 need citation; Line 152: please add NeuN antibody company and catalogue number.

**Response:** We have revised the wording, added the requested citation, and included the NeuN antibody company and catalogue number in the Methods section.

July 15, 2026

RE: Life Science Alliance Manuscript #LSA-2026-03677RR

Prof. Manuel Alexander Friese
University Medical Center Hamburg-Eppendorf
Institute of Neuroimmunology and Multiple Sclerosis
Falkenried 94
Hamburg D-20251
Germany

Dear Dr. Friese,

Thank you for submitting your Research Article entitled "The transcriptional cortical response to daily torpor in Phodopus sungorus". It is a pleasure to let you know that your manuscript is now accepted for publication in Life Science Alliance. Congratulations on this interesting work.

The final published version of your manuscript will be deposited by us to PubMed Central upon publication.

Your manuscript will now progress through copyediting and proofing. It is journal policy that authors provide original data upon request.

Your article will publish open access upon publication under a CC-BY license.

Reviews, decision letters, and point-by-point responses associated with peer-review at Life Science Alliance will be published, alongside the manuscript. If you do want to opt out of having the reviewer reports and your point-by-point responses displayed, please let us know immediately.

***IMPORTANT: If you will be unreachable at any time, please provide us with the email address of an alternate author. Failure to respond to routine queries may lead to unavoidable delays in publication.***

Scheduling details will be available from our production department. You will receive proofs shortly before the publication date. Only essential corrections can be made at the proof stage so if there are any minor final changes you wish to make to the manuscript, please let the journal office know now.

DISTRIBUTION OF MATERIALS:
Authors are required to distribute freely any materials used in experiments published in Life Science Alliance. Authors are encouraged to deposit materials used in their studies to the appropriate repositories for distribution to researchers.

You can contact the journal office with any questions, contact@life-science-alliance.org

Again, congratulations on a very nice paper. I hope you found the review process to be constructive and are pleased with how the manuscript was handled editorially. We look forward to future exciting submissions from your lab.

Sincerely,

Sarita Hebbar, PhD
Scientific Editor
Life Science Alliance
http://www.lsajournal.org
